# The Middle Cambrian fossil *Pikaia* and the evolution of chordate swimming

**DOI:** 10.1186/2041-9139-3-12

**Published:** 2012-07-06

**Authors:** Thurston Lacalli

**Affiliations:** 1 Biology Department, University of Victoria, Cunningham Building, Victoria, BC, V8W-3N5, Canada

**Keywords:** *Pikaia*, Basal chordates, Myomere and notochord evolution, Amphioxus, Swimming mechanics, Vertebrate muscle fibers, Yunnanozoans

## Abstract

Conway Morris and Caron (2012) have recently published an account of virtually all the available information on *Pikaia gracilens*, a well-known Cambrian fossil and supposed basal chordate, and propose on this basis some new ideas about *Pikaia*’s anatomy and evolutionary significance. Chief among its chordate-like features are the putative myomeres, a regular series of vertical bands that extends the length of the body. These differ from the myomeres of living chordates in that boundaries between them (the myosepta) are gently curved, with minimal overlap, whereas amphioxus and vertebrates have strongly overlapping V- and W-shaped myomeres. The implication, on biomechanical grounds, is that myomeres in *Pikaia* exerted much less tension on the myosepta, so the animal would have been incapable of swimming as rapidly as living chordates operating in the fast-twitch mode used for escape and attack. *Pikaia* either lacked the fast-twitch fibers necessary for such speeds, having instead only slow-twitch fibers, or it had an ancestral fiber type with functional capabilities more like modern slow fibers than fast ones. The first option is supported by the sequence of development in zebrafish, where both myoseptum formation and fast fiber deployment show a dependence on slow fibers, which develop first. For *Pikaia*, the absence of fast fibers has both behavioral and anatomical implications, which are discussed. Among the latter is the possibility that a notochord may not have been needed as a primary stiffening device if other structures (for example, the dorsal organ) could perform that role.

## Background

**Figure 1:**
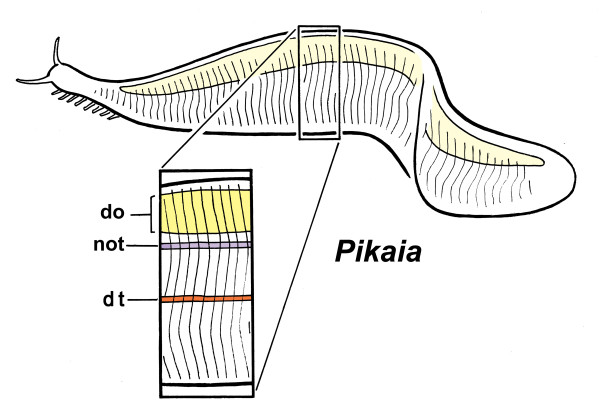
***Pikaia gracilens*, as reconstructed by Conway Morris and Caron**[1].The head bears a pair of tentacles, probably sensory in nature, and paired rows of ventrolateral projections that may be gills. Not shown: the expanded anterior (pharyngeal) region of the digestive tract, and the dorsal shield-like structure, the anterior dorsal unit, that lies above it. The boxed detail shows the main axial features: the dorsal organ (do), and the putative notochord (not) and digestive tract (dt). The size range among specimens is 1.5 to 6 cm, which makes this animal very close in size to the adult stage of modern lancelets (amphioxus)

Zoology texts typically list four diagnostic features of chordates: pharyngeal (that is, gill) slits or pores, a notochord, a dorsal nerve cord and serial (or segmental) muscles. This last feature, represented by the somite-derived myomere series in the case of cephalochordates (amphioxus) and vertebrates, is the subject of this account, stimulated by the recent description [1] by Simon Conway Morris and Jean-Bernard Caron (here referred to as CMC) of the Middle Cambrian fossil *Pikaia gracilens* (Figure 1) from the Burgess Shale of British Columbia. The authors interpret *Pikaia* as a basal chordate and, though this conclusion is provisional, it would be perverse to deny the key similarities between this animal and what would be expected of a basal chordate: much of the body is occupied by a series of vertical bands resembling the septa between segmental muscles, and the authors identify an axial trace that could be either a notochord or a notochord and nerve cord combined. In addition, however, there are peculiar features not known from living chordates: a sausage-shaped dorsal organ running the length of the trunk, and an anterior shield-like structure, the anterior dorsal unit, covering the head region. *Pikaia* does not, therefore, fit entirely comfortably with modern chordates, suggesting that it is either divergent, if it is a chordate, or is a basal member of the chordate lineage differing in significant ways from surviving members of that lineage.

Of the chordate features listed above, the first, the pharyngeal slits, have an evolutionary history that probably predates chordates by a considerable interval, because apparently homologous structures occur in more basal deuterostome phyla, in living hemichordates and fossil echinoderms [2-4]. Pharyngeal pores or slits, where they occur, are assumed to play an ancestral role in deposit- or filter-feeding as a means for disposing of excess water entering the mouth and pharynx with food particles. The remaining three features mentioned above, which together constitute the axial complex, are responsible for undulatory swimming. These are considered to be restricted to chordates alone, and form a suite of characters that are closely linked anatomically, and that operate functionally as a unit [4]. Of the three, only the notochord has a plausible counterpart, the enteropneust stomochord, in more basal deuterostomes, but the two structures are not currently considered to be homologous [5]. We thus have very little evidence from comparative studies to indicate how the axial complex evolved, all at once or step by step, and if the latter, what the key steps might have been. CMC have made a start, in their paper, by discussing the role the dorsal organ may play in antagonizing the force of muscle contraction during swimming, at a stage in chordate evolution when the notochord might not yet have fully taken over this function. This commentary on their paper is an attempt to carry the argument somewhat further, using simple biomechanical principles to clarify the relation between myomere morphology and swimming mode, and to discuss some of the implications this has for the behavior and mode of life of *Pikaia* and its kin.

## Myomere shape and its implications

CMC discuss myomere shape at some length because of significant ways this differs between *Pikaia* and modern chordates. Myomeres in *Pikaia* are taller and narrow (box, Figure 1), while the myosepta, which form the interface between them, are gently curved, or sigmoidal, such that the overlap between adjacent myomeres nowhere exceeds more than about one segment. CMC discuss this in relation to the much more angled V- and W-shaped myomeres of amphioxus and vertebrates, but there is more to be drawn from this comparison in my view. Tunicate larvae are omitted from both CMC and this account because, based on current phylogenies [6], both they and their tail musculature are reduced secondarily to a degree that makes them uninformative regarding the nature of ancestral myomeres.

Vertebrates, as one would expect, have the most complicated segmental architecture. Myomeres in adult fishes are substantial masses of tissue whose thickness seriously complicates the task of analyzing the internal stresses generated during swimming. This is chiefly because the tension exerted on the spinal axis as a given fiber contracts varies with the distance that fiber lies from the central axis. To avoid producing unequal stresses across the myomere, the fibers are arranged helically and the myosepta have a complex three-dimensional shape approximating a W [7,8]. The accepted explanation, which requires some fairly sophisticated modeling [9], is that this arrangement minimizes the difference in tension experienced on the opposing faces of each myoseptum as a means of reducing energy loss.

**Figure 2:**
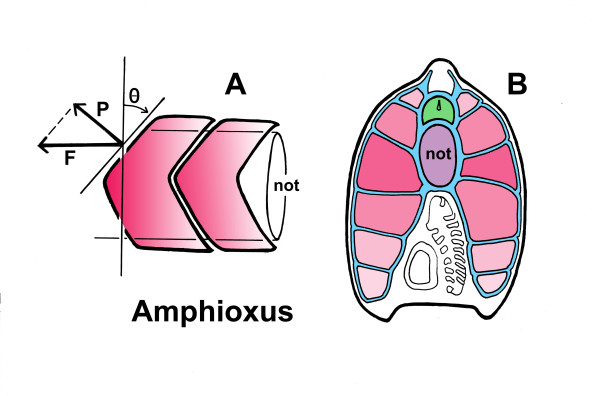
**The beneficial consequences of having chevron- or V-shaped myomeres, illustrated using amphioxus. (A)** The V shape guarantees that the force acting perpendicular to the myoseptum (vector P) is less than the force of contraction (vector F) by an amount that increases with increasing angle (Θ) to the vertical. The degree of incline shown is typical for amphioxus larvae, and increases with increasing age. **(B)** Somite overlap in young adult amphioxus, modified from [11]. The central components of the locomotory system are the notochord (not, shown in violet) and the nerve cord (green). These are bound to the myomeres (pink) by sheaths of basal lamina (blue). The V-shaped myomeres are positioned so that the tip of the caudal-most in any section is adjacent to the notochord, while the extended tails of progressively more anterior myomeres (shown in progressively lighter shades, compare with A) are ranged above and below. Because every point along the notochord has essentially the same complement of septa, this arrangement ensures that the force of contraction experienced by the notochord is distributed evenly along its anteroposterior axis, rather than being borne at specific sites

Myomeres in larval fish and in amphioxus are chevron- or V-shaped and are much less massive. They are flattened mediolaterally and so, in effect, can be treated as a first approximation as flat, two-dimensional structures. This makes it much easier to understand the biomechanical issues involved without resorting to a detailed mathematical and computer analysis. Muscle fibers in amphioxus attach to the myosepta directly [10] rather than to the notochord sheath, so the greatest stresses on the notochord are borne where the septa join the sheath. But the whole complex is also bound tightly together by sheets of basal lamina, and therefore acts as a single unit, as opposed to relying on an array of subsidiary tendons and bony struts to distribute the forces, as in fish. So, using amphioxus as a model, what are the advantages of having V-shaped myosepta rather than vertical ones? The key seems to be in the angle of inclination, that is, the steepness of the V. Because the muscle fibers are aligned horizontally, along the body axis, it is in that direction that force is exerted during contraction. Expressed as vectors (Figure 2A), this force has two components, one directed perpendicular to the anterior surface of the myomere (P in the figure), the other parallel to it (along the dashed line). The former is the more important component, as stresses in that direction are the ones most likely to result in mechanical failure of the septa, for example, by tearing the myomeres apart, whereas forces acting parallel to the septa would only be important if damage by shear were the key issue. From simple trigonometry, the perpendicular component of force is less than the total force of contraction in proportion to the cosine of the angle the myoseptum makes with the vertical: for given force of contraction, increasing this angle (Θ in the figure) decreases the stresses on the myoseptum. For amphioxus, from my own material and published photos [11], the measured incline is in the range of 40 to 55° in larvae, depending on stage, and 64 to 68° for adults. This translates into a reduction of force by roughly 24 to 43% in larvae and 56 to 62% in adults. This is a significant benefit if, in consequence, less is required by way of reinforcement to the enclosing system of sheath and septa.

There is another advantage in arranging for the boundaries between myomeres to be steeply inclined: it maximizes the overlap between myomeres and so distributes the force of contraction more evenly along the length of the notochord. To illustrate this, consider an alternative, of straight myosepta transversely oriented more or less vertically, as in *Pikaia*. If the muscles in such an animal attach only at the septa (as they do in amphioxus), all the force of contraction would be borne at the sites where these join the notochord sheath. Along the sheath, therefore, sites of maximum and minimum stress would alternate, so there is both a greater risk of catastrophic failure (for example, by kinking) and less likelihood that the structure as a whole will flex smoothly. In contrast, when septa are inclined to allow for multiple overlaps, every point along the notochord bears essentially the same load as measured by the number of septa attaching to it (Figure 2B). This evens out the stress due to contraction. In amphioxus, the overlap ranges from one to two segments in larvae to three to four in the adult, considerably more in the latter than in *Pikaia* for an animal of approximately similar size.

## Multiple locomotory modes and escape from predators

The implication of the steeper angle of incline of amphioxus and vertebrate myosepta, in comparison with *Pikaia*, is that the forces generated by myomere contraction are a more serious issue for amphioxus and fish, requiring this adaptation, than for *Pikaia*. It is thus reasonable to suppose that *Pikaia* myomeres would not have been capable of exerting as much force as those of modern chordates, and consequently peak swimming speed in *Pikaia* would have been considerably less. There is an assumption here, as the conclusion depends on the supposition that angling of myosepta gives a direct measure of the force of contraction across a range of taxa, both living and long extinct. For this to be true, the angle of inclination must be a comparatively plastic feature in evolutionary terms, one that is able to adapt rapidly in any lineage when it is advantageous to do so. This may be reasonable because, as structural features go, myomere shape is one that in principle should be easy to adjust incrementally. Indeed, myomeres in nearly contemporaneous vertebrates such as *Haikouichthys*[12], which are roughly intermediate in shape between those of *Pikaia* and amphioxus, show an incremental gradation in shape along the body. Those in the caudal region are more steeply inclined, which is precisely where one would predict the greatest stress if, for example, the animals had an escape response involving powerful tail flips.

There is a further issue that needs to be considered in this context, and that is the nature of the muscle fibers themselves. Myomeres in amphioxus and fish have basically two fiber types with quite different functional capabilities [13,14]. Rapid, episodic burst swimming results from the contraction of fast (or fast-twitch, or deep) fibers, of which the bulk of the myotome is composed. In addition, however, there are sets of slow (slow-twitch, or superficial) fibers along the lateral margin of the myomere, fibers that do not fatigue as quickly and are responsible for prolonged bouts of slower swimming. These are used for long distance swimming (for example, cruising or migration) in fishes, for diurnal vertical migrations in the water column in amphioxus larvae, and to adjust position in burrows in adult amphioxus. With regard to amphioxus specifically, there is a lingering misconception that the burrowing habit has resulted in its becoming a rather poor and ineffective swimmer. The contrary is, in fact, the case, as anyone who has tried to net an amphioxus swimming at peak speed will attest. The animal is mass of muscle and connective tissue capable of powerful writhing movements when restrained, and of considerable speed when swimming, greater than many fish when adjusted for relative body size [15]. In part this is possible because, lacking eyes, amphioxus need not look where it is going. Instead, and because of its tough cuticle, it simply bounces off obstacles in its path. Such behaviors are due to the action of the fast fibers, and are well described in previous accounts of the behavior of larvae [16,17] and adults [18]. Clearly, a much greater physical stress is being exerted on the support structures of the body during fast than slow swimming, which must certainly be a factor in explaining the adaptive advantages of V-shaped myotomes.

The conclusion one can draw from this is, that because *Pikaia* lacks steeply inclined myosepta, its muscle fibers have properties more like slow fibers than fast ones. If it could be shown that the slow system evolved first, *Pikaia* could quite logically be interpreted as representing a stage in chordate evolution before the evolution of fast fibers. With some further biomechanical analysis of contraction strengths, septum thickness, and so on, a good deal could probably be inferred about the behavioral capabilities and mode of life of *Pikaia* based on the known properties of modern slow fibers. However, the evidence for a sequence in the evolution of fiber type, of slow before fast or vice versa, is at best circumstantial. Logically, one might suppose that the less effective locomotory mode would have evolved first, with subsequent improvements being a response to extraordinary new adaptive pressures. Since the Lower to Middle Cambrian was a period when fast-swimming predators with high-resolution eyes were appearing (chiefly the anomalocarids and their kin [19]), slow modes of swimming that would have been adequate for basal chordates nearer the dawn of the Cambrian would have been increasingly less so. Faster modes would have had to evolve. That this happened quickly is evident from the vertebrates of the time, for example, *Haikouichthys*, as mentioned above, which predates *Pikaia* by 5 million years at least. Conodonts, though much later, also have this feature [20,21], as befits another putative basal vertebrate. Both belong to lineages that survived the Cambrian, whereas *Pikaia* evidently, so far as we know, did not.

The developmental sequence is suggestive here as well. In fish (from zebrafish data), the slow fibers develop first, and then migrate to the outer surface of the somite [22,23]. The fast fibers develop later, but depend on cues from the slow fibers for correct deployment [24], while myoseptum formation is also impaired in the absence of slow fibers [25]. That slow fibers are so fundamentally important to the normal sequence of developmental events is certainly consistent with an early origin, possibly predating the evolution of the later developing fast fiber system, but there are other interpretations (see below). Further evidence for the relative antiquity of the slow system, again circumstantial, comes from amphioxus, where there are significant differences in the way the two systems are innervated. From data on larvae [17,26] fast fibers are innervated by neurons distributed along the nerve cord via conventional synapses, and mechanosensory input is routed to this system alone as befits an escape pathway. The slow system, in contrast, is innervated by a dedicated series of neurons in the anterior nerve cord via a less specialized type of synaptic contact. The principle inputs, so far as this has been determined, is from neurons in the cerebral vesicle, specifically in the amphioxus homolog of the hypothalamus, and from the dorsal ocelli (eyespots). In both the hypothalamic and ocellar pathways, paracrine release from large terminals predominates over specialized synapses. The former, where it occurs (for example, in core limbic elements of the vertebrate brain), is generally considered an indication of evolutionary antiquity [27]. The involvement of the ocelli in the slow circuits may also be significant, since response to light both during vertical migration (in larvae) and in burrows (adults) are aspects of feeding behavior, and hence sufficiently basic to survival that they probably predate the rise of visual predators. In addition, because the neurotransmitter in these photoreceptors is related to a gonadotropin-releasing hormone [28], there is a possibility that the ancestral function of the slow system was in some way involved with reproduction or mating behavior.

While the above is consistent with the slow system being evolutionarily older, there is an alternative suggested by molecular data on fiber type specification: that the fast fibers are the default state of myocyte development, while the slow fibers follow a divergent pathway initiated by early Hedgehog signaling from the notochord [29]. This would imply that slow fibers, at least in their modern form, are a late addition to an older program of myocyte differentiation. However, even if the mechanism controlling fast fiber differentiation is an ancient one, it does not mean that the ancestral fibers matched modern fast fibers in their functional capabilities. Quite the converse, since on the evidence of myomere shape in *Pikaia*, it would seem that the forces generated by the ancestral fibers were much less, probably more like modern slow fibers than fast ones. Suppositions concerning the behavior and mode of life of *Pikaia* need then to be assessed with this in mind.

## General anatomy and mode of life: some conjectures

If one assumes that *Pikaia* was incapable of a fast escape from visual predators of the *Anomalocaris* type, a strategy of avoidance would likely be its preferred solution. *Pikaia* was probably not, therefore, an animal that spent a lot of time high up in the water column or near the surface during daylight hours. One could envisage it feeding inconspicuously near the bottom, perhaps cruising along and browsing on benthic detritus or microbial mats, directed by its paired sensory tentacles. This accords with the conclusions of CMC that, from the mode of preservation, *Pikaia* was likely epibenthic rather than fully pelagic. Alternatively, it may have migrated vertically to spend time feeding at the surface, but only at night. Unfortunately, as the apparently tiny mouth is difficult to interpret in the fossils, there is little direct evidence from the morphology as to how *Pikaia* fed. Because the mouth was not large, it seems unlikely that *Pikaia* took in large volumes of water when feeding, however, as a suspension feeder normally would be expected to do.

The series of paired appendages projecting from the pharyngeal region may be gills, and there are indications in some specimens of small pores at their bases. The presence of projecting external gills rather than slits would imply that the oxygen demand of even slow swimming required them. This is perhaps surprising given the flattened cross-sectional profile of the body, as it has considerable surface area for respiration across the skin alone. This is evidently sufficient for amphioxus, where gas exchange is predominantly across the general surface of the body [30]. A need to expand the gas exchange surface further in *Pikaia* could be an indication that it experienced severe anoxia, at least periodically, due to its habitat. There is a further problem in the putative gills being located so far from the bulk of the trunk musculature. An extensive vascular system would then be needed to distribute oxygenated blood to the trunk, so vascular traces should be expected in suitably preserved material. CMC in fact identify one such trace, of what may be a ventral blood vessel, but this begs the question of why there are no indications of vessels needed to complete the circuit. On comparative anatomical grounds, these should lie above the alimentary canal in a situation comparable to the chordate dorsal aorta.

The notochord and nerve cord, expected as parallel axial traces, pose a different problem, because there is no *a priori* reason to suppose that they should have the same close linkage with the muscles as in modern chordates. Undulatory swimming is accomplished without a notochord in diverse taxa, ranging from small (fluke cercariae) to large (segmented annelids, for example, *Nereis*, some species of which attain lengths of more than a meter). In chordates, the notochord acts as a compression strut, resisting the force of contraction during swimming. The need for this would be especially acute during rapid escape swimming, but much less so for an animal capable only of slow swimming. This is not to say that *Pikaia* would not have had a notochord (though this cannot be ruled out), only that it need not have been the core structure around which the myomeres are organized. Any number of other axial structures might have played this role either alone or in concert with a notochord. The dorsal organ is the relevant axial structure here, as it may have been some kind of turgid sac or rod. Viewed from the perspective of modern chordates, few zoologists would accept, given its extreme dorsal location, that such a structure would be an effective device for attaching myomeres and antagonizing their contraction. As CMC point out, however, and discuss at some length, a truly basal chordate might well have had a provisional and less efficient support system prior to the evolving one dependent on the notochord alone. The dorsal organ could have been adequate for this purpose. Myomeres would attach along the sides of the dorsal organ and extend ventrally from it, probably attached to a midsagittal sheet (or sheets) of connective tissue. Waves of contraction passing along the body would have least amplitude at the level of the dorsal organ, and become progressively more pronounced ventrally as distance from the dorsal organ increased. The undulations produced would thus be graded along the dorsoventral axis, from shallow dorsally to deeper ventrally. A notochord could add accessory stiffening at roughly the level of the gut, which may explain why it derives embryologically from the same rudiment as the latter. Indeed, as locomotory capabilities of early chordates improved, there might have been an increasing need for such a stiffening device to damp undulations that would otherwise impair the movement of the products of digestion along the digestive tract. A notochord that evolved initially for this purpose would be well placed to later take on a more central role in body support as the need arose.

As CMC also point out, having the dorsal organ act in this fashion links *Pikaia* to another group of puzzling fossils of Cambrian age, the yunnanozoans. These are considered by many to be chordates or close to them [31,32], but there are other possibilities [33-35]. Yunnanozoans have a dorsal sail-like structure that is apparently cuticularized and at least partially articulated. Muscle fibers have been reported in association with this structure [31], though this interpretation is contentious, but the very fact of articulation implies an ability to flex due to the action of underlying muscles. Assuming this is a valid interpretation, yunnanozoans provide a second example of axial support in a putative chordate by a dorsal structure that is not a notochord. With a second structure available to share the load, the ancestral notochord would have been freed of at least some of the constraints that determine its size, robustness and location in modern forms. The fossils need to be interpreted with this in mind.

## Conclusions

The nearly vertical junctions between myomeres in the putative basal chordate *Pikaia gracilens* are shown to be indicative of a muscle fiber type more similar to the slow fibers of modern chordates than fast fibers. It is not known if the former evolved first, but if so, *Pikaia* may represent a stage in evolution before chordates had fast fiber systems. If not, then *Pikaia* was nevertheless probably limited to a relatively slow form of locomotion. This has implications for the mode of life, implying passive avoidance rather than fast escape from predators, and for the anatomy, in that less stiffening of the body is needed to oppose the contractions of the axial musculature. This last point could explain the prominence of the dorsal organ in *Pikaia*, as an alternative support structure at a time when the notochord played a more limited role in locomotion.

## Competing interests

There are no competing interests.
